# Exploring the Variability in Phenolic Compounds and Antioxidant Capacity in Olive Oil By-Products: A Path to Sustainable Valorization

**DOI:** 10.3390/antiox13121470

**Published:** 2024-11-29

**Authors:** Jessica Paié-Ribeiro, Filipa Baptista, Maria José Gomes, Alfredo Teixeira, Victor Pinheiro, Divanildo Outor-Monteiro, Ana Novo Barros

**Affiliations:** 1 Animal Science Department, University of Trás-os-Montes e Alto Douro (UTAD), 5000-801 Vila Real, Portugal; mjmg@utad.pt (M.J.G.); vpinheir@utad.pt (V.P.); divanildo@utad.pt (D.O.-M.); 2 Veterinary and Animal Research Centre (CECAV), University of Trás-os-Montes e Alto Douro, 5000-801 Vila Real, Portugal; 3 Centre for the Research and Technology of Agro-Environmental and Biological Sciences, CITAB, University de Trás-os-Montes e Alto Douro (UTAD), 5000-801 Vila Real, Portugal; fbaptista@utad.pt; 4 AL4animals, Quinta de Prados, 5000-801 Vila Real, Portugal; 5 Mountain Research Center (CIMO), Polytechnic Instituto of Bragança, Campus de Santa Apolónia, 5300-253 Bragança, Portugal; teixeira@ipb.pt

**Keywords:** olive cake, food waste valorization, bioactive compounds, antioxidant capacity, nutraceuticals

## Abstract

The olive oil industry generates large volumes of by-products, creating notable environmental and economic concerns. Among these, olive cake (OC)—a primary by-product of olive oil extraction—stands out due to its high content of bioactive compounds and potential for value-added recycling. This study focused on characterizing six OC samples from the Trás-os-Montes and Alto Douro regions, collected at different processing times and mills. The samples included two derived from pressing (COC), two from two-phase centrifugation (TPOC; one partially pitted and one dehydrated), and two exhausted OC (EOC) samples. Fundamental analyses assessed total phenols, *ortho*-diphenols, flavonoids, antioxidant capacity, and tannin content. Results revealed significant variation (*p* < 0.05) in phenolic composition, namely *ortho*-diphenols and flavonoid levels among the samples. EOC 2 exhibited the highest concentrations (19.61, 21.82, and 20.12 mg CAT/g, respectively), while COC 2 had the lowest (5.08, 5.08, and 2.76 mg GA/g, respectively). This correlated with elevated antioxidant activity in EOC 2, as measured by FRAP, DPPH, and ABTS assays (129.98, 78.00, and 56.65 μmol Trolox/g). In contrast, COC 1 and COC 2 displayed the lowest antioxidant activities (32.61 μmol Trolox/g in FRAP and 17.24 and 18.98 μmol Trolox/g in DPPH). Tannin analysis showed the highest total tannin content in the dehydrated and pitted OC samples (250.31 and 240.89 mg CAT/100 g), with COC 2 showing the lowest (88.17 mg CAT/100 g). Condensed tannin content varied significantly, with EOC 2 presenting the highest level (328.17 mg CAT/100 g) and COC 2 the lowest one (20.56 mg CAT/100 g). Through HPLC-PDA-MS, 22 compounds were identified, with luteolin and verbascoside being particularly prevalent. This in-depth characterization supports the potential valorization of olive by-products, advancing sustainability and promoting a circular economy in the olive oil sector.

## 1. Introduction

In Europe, food waste reaches alarming levels, with around 88 million tons discarded annually [1]. This situation represents a substantial social and environmental challenge and contradicts the principles of sustainable food production and consumption. However, a new perspective is emerging; instead of being discarded, some waste and by-products from the food industry can be converted into valuable nutrient sources, offering promising solutions for addressing the social and health challenges facing humanity in the 21st century [2].

The olive tree (*Olea europaea* L.) is a dicotyledonous angiosperm tree that belongs to the *Oleaceae* family. Native to Asia Minor and Syria, the olive tree is now widely cultivated across the Mediterranean region. Currently, Spain, Italy, and Greece are the top producers of olives and olive oil [3,4,5,6]. Each year, millions of tons of by-products, including olive pomace, branches, leaves, and pits (skins and seeds), are generated during pruning, harvesting, and producing olive oil [7].

Olive oil is typically produced using three methods of extraction, which include the traditional pressing process and a three-phase and two-phase centrifugal extraction system [2,8,9]. Although traditional pressing is relatively outdated, it persists in some olive oil mills. In this method, grinding the olives generates a semi-solid mass subjected to pressure to extract the oil. This results in a solid fraction (olive skin) and an emulsion containing the oil and water, separated by decantation [10,11,12]. The three-phase system results in three fractions at the end of the process, namely a solid (olive skin or pomace) and two liquids (oil and wastewater). While this system has advantages over traditional pressing—such as full automation, improved oil quality, and a smaller spatial footprint—it also has some drawbacks. These include higher water and energy consumption, increased wastewater production, and more expensive installations [13].

The “two-phase” system was developed at the end of the 1991/1992 olive oil campaign. Due to its reduced water consumption, it was introduced to the market as an “ecological” option. This method produces two final products, namely a solid fraction known as *alperujo* (which includes wet olive skin and wet pomace) and a liquid fraction of olive oil. The two-phase system quickly replaced the traditional three-phase method. Additionally, the olive cake from the three-phase system can undergo a second extraction of residual oil using organic solvents after it has been dried [14,15,16].

Managing these by-products represents a growing producer cost and a global environmental challenge. Despite their potential use in animal feed or as fuel, they are often discarded, burnt, crushed, and scattered in the environment [17].

The composition of OC varies based on climate and extraction methods. It typically contains the pit (18–32%), pulp, shell, kernel, residual oil, and approximately 40–60% water. Olive cake is also a rich source of beneficial molecules, such as peptides, flavonoids (quercetin), tocopherols, and other polyphenols. Notably, it contains vitamin E, a group of exogenous antioxidants that includes tocopherols. These lipophilic molecules can synergize with phenolic acids, enhancing the antioxidant action of olive cake [11,18,19]. Additionally, this antioxidant activity demonstrates a broad spectrum of positive biological effects on health, including antigenotoxic, cytotoxic, anti-allergic, antimicrobial, cardioprotective, and anti-inflammatory properties [20,21,22,23,24].

The composition of phenolic compounds differs greatly among various tissues of the olive tree, such as leaves, branches, and small twigs, as well as its different by-products from olive oil production [17]. Among olive cake’s most abundant phenolic compounds are hydroxytyrosol, its derivatives, and oleuropein; however, oleuropein is found in higher concentrations in other parts of the olive. Other significant compounds include verbascoside, rutin, caffeoylquinic acid, luteolin-4-glucoside, and oleoside derivatives [17,25,26]. The challenge of disposing of OC continues, emphasizing the need to explore new technologies for its profitable and sustainable use. Studies have demonstrated that phenolic extracts from olive cake possess high antioxidant activity, indicating their potential as additives in the food industry. Notably, only around 2% of the olives’ phenolic compounds are transferred to the final product during the production of virgin olive oil, leaving the remaining 98% in the olive cake [27]. This discovery opens the door to utilizing olive cake, transforming a by-product into a valuable ingredient with beneficial health properties [28,29,30].

The valorization of OC, a by-product of olive oil production, can significantly reduce carbon footprints through various methods, such as producing activated carbon (AC) and generating clean energy. Furthermore, considering the need to deepen knowledge about the phenolic variability of OC, this work takes an innovative approach by investigating the bioactive compounds present in this by-product individually [31]. Although the literature has already demonstrated the richness of total phenols in olive oil production residues, the isolated quantification of specific compounds has received less attention. The identification and detailed characterization of these biphenols are crucial to understand the potential of each molecule [31]. In scenarios aimed at recovering specific compounds for industrial and commercial use, OC stands out as a promising source due to its bioactive compounds. In the food industry, OC extracts can be used as natural antioxidants in meat products, which can help enhance product longevity and minimize food waste [31,32].

Furthermore, in animal health, supplementing feed with these extracts can contribute to stabilizing physiological conditions, reducing oxidative stress and improving intestinal health [33]. In the nutraceuticals sector, OC extracts can be used to produce enriched bioactive food ingredients, promoting health benefits for consumers and expanding the possibilities for applying these compounds [34]. This descriptive approach allows us to broaden our knowledge of the chemical composition of OC and identify molecules with more excellent added value, in line with the circular economy’s demands and sustainability principles.

This study aimed to investigate various olive cakes’ chemical composition and determine their total tannin (TT) and condensed tannin (CT) content. Additionally, we sought to assess the levels of total phenolics and the antioxidant capacity of different OC extracts. We also aimed to identify the individual phenolic compounds responsible for this antioxidant activity using high-performance liquid chromatography coupled with mass spectrometry (HPLC-PDA-MS).

## 2. Materials and Methods

### 2.1. Chemicals and Reagents

The following compounds were used in this study: gallic acid, sodium molybdate, sodium carbonate, sodium acetate, catechin, potassium persulfate, and methanol. The following chemical reagents were used: 2,2′-Azino-bis(3-ethylbenzothiazoline-6-sulfonic acid) diammonium salt (ABTS•+), 6-Hydroxy-2,5,7,8-tetramethylchromone-2-carboxylic acid (Trolox), and 2,2-diphenyl-1-picrylhydrazyl (DPPH•). They were obtained from Sigma-Aldrich (Steinheim, Germany).

Folin–Ciocalteu’s reagent, formic acid (pro-analysis), and acetonitrile (HPLC gradient grade) were purchased from Panreac Química S.L.U. (Barcelona, Spain). Ultrapure water was prepared using a Millipore water purification system.

Additionally, aluminum chloride, sodium nitrite, and sodium hydroxide were sourced from Merck (Darmstadt, Germany). Methyl cellulose solution and ammonium sulfate were also acquired from Sigma-Aldrich (Saint Louis, MO, USA). Hydrochloric acid and vanillin were obtained from Merck Chemicals (Darmstadt, Germany).

### 2.2. Samples of Olive Cakes

In this study, six types of olive cakes (OCs) were collected on different dates from various mills in the Trás-os-Montes and Alto Douro regions. The collection included two olive cakes obtained through pressing (COC), two olive cakes from a two-phase centrifugation process—one of which was partially pitted and the other dehydrated (TPOC)—and two exhausted olive cakes (EOCs). The chemical characterization of each type of OC was previously described by Paié-Ribeiro et al. [29]. The samples were gathered during the 2020/21 olive oil production season and stored at −20 °C until subsequent analysis.

### 2.3. Phenolic Extract Preparation

The analyses performed for polyphenolic determination were based on the content of total phenols, *ortho*-diphenols, and flavonoids. For all analyses, three replicates (*n* = 3) were performed in 96-well microplates (Frilabo, Milheirós, Portugal), and the absorbance measurements were performed with Multiskan equipment (Thermo Fisher Scientific^®^, Vantaa, Finland).

To determine the total phenolic content (TPC), 40 mg of each OC sample was added to 1.5 mL of a 70% (*v*/*v*) ethanol solution. The choice of 70% ethanol was based on evidence from the literature that highlights this concentration as ideal for maximizing the extraction of phenolic compounds [35]. Hydroalcoholic solutions with this proportion balance the polarity of the solvent, allowing for an efficient extraction of both hydrophilic and lipophilic compounds [36,37].

**Figure 1:**
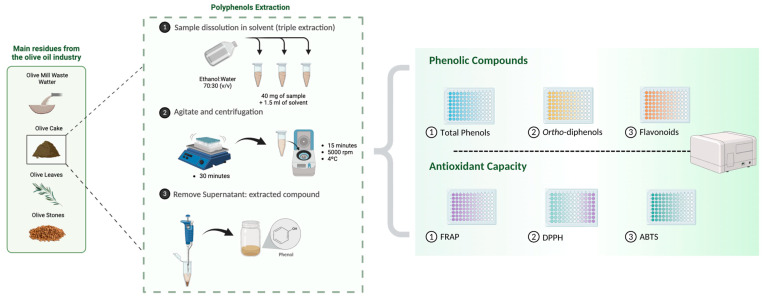
Schematic representation of the methodology used to extract and quantify polyphenols (total phenols, *ortho*-diphenols, and flavonoids) and antioxidant capacity (FRAP, DPPH, and ABTS) in olive cake.

The mixture was stirred in a vortex for 30 min at room temperature to increase solubility and facilitate the extraction of the phenolic compounds. The mixture was then centrifuged at 5000 rpm for 15 min at 4 °C using a Sigma centrifuge (Steinheim, Germany). The supernatant containing the extracted compounds was carefully separated from the solid residue and the accumulated volume was adjusted to 5 mL, preserving the initial composition of the solvent to ensure consistency between the samples. This extraction procedure was repeated three times, and the supernatants from each stage were combined (Figure 1).

### 2.4. Determination of Phenolic Content

The total polyphenol content of the extracts was determined using the Folin–Ciocalteu reagent, as described by Yu et al. [38]. In brief, 20 μL of a gallic acid standard, sample, or distilled water (as a blank) was added to each well of a 96-well microplate. Next, 100 μL of Folin–Ciocalteu reagent previously diluted in water (1:10) and 80 μL of 7.5% sodium carbonate solution were added. After a 30 min incubation at 40–45 °C, the absorbance was measured at 725 nm. The results were expressed in milligrams of gallic acid equivalent per gram of sample (mg GA g^−1^). This method involves the formation of a blue-colored complex initiated by the reduction of phosphomolybdate–phosphotungstate by phenolic compounds.

For the calibration curve and regression equation (y = 0.0047 ± 0.0899; R^2^ = 0.996), gallic acid solutions were prepared at concentrations of 200, 150, 100, 75, 50, 25, 10, 5, and 2.5 mg L^−1^ using a stock solution of gallic acid at 250 mg L^−1^.

#### 2.4.1. *Ortho*-Diphenol Content

The *ortho*-diphenol content (ODC) was determined following the procedure outlined by Yu et al. [38]. As in the previous method, gallic acid was used as the standard for the calibration curve construction, which covered a concentration range from 5 to 200 mg per gram of solution. A total of 160 μL of a sample, standard, or distilled water (blank) was added to each well of the microplate, followed by 40 μL of sodium molybdate solution. After incubating for 15 min, the absorbance was recorded at 375 nm.

For the calibration curve and regression equation, gallic acid solutions (250 mg L^−1^) were prepared at concentrations of 200, 150, 100, 75, 50, 25, 10, 5, and 2.5 mg L^−1^ (y = 0.0051 ± 0.0844; R^2^ = 0.0994). The *ortho*-diphenol content was then expressed as milligrams of gallic acid equivalent per gram of sample (mg GA g^−1^).

#### 2.4.2. Flavonoid Content

The flavonoid content (FC) was assessed using the colorimetric method outlined by Yu et al. [38]. In summary, 24 μL of a standard, sample, or distilled water (blank) and 28 μL of sodium nitrite (NaNO_2_) solution were added to a microplate. After 5 min incubation, 28 μL of aluminum chloride (AlCl_3_) solution was introduced. Following an additional 6 min of incubation, 120 μL of sodium hydroxide (NaOH) solution was added. The microplate was then gently stirred for 30 s, and the absorbance was recorded at 510 nm.

For the calibration curve and regression equation, catechin solutions (250 mg L^−1^) were prepared at concentrations of 200, 150, 100, 75, 50, 25, 10, 5, and 2.5 mg L^−1^ (y = 0.0017 ± 0.0516; R^2^ = 0.998). The FC was expressed as milligrams of catechin equivalent per gram of sample (mg CAT g^−1^).

### 2.5. Determination of Antioxidant Capacity

The analyses conducted to determine antioxidant capacity included DPPH, ABTS, and FRAP assays. All assays were performed in triplicate (*n* = 3) using 96-well microplates (Frilabo, Milheirós, Portugal) with absorbance measurements taken using Multiskan equipment (Thermo Fisher Scientific^®^, Vantaa, Finland). These antioxidant capacity assays also used the extracts utilized for phenolic composition analysis and Trolox as a standard. To ensure that the extract was suitable for antioxidant capacity analysis, the polarity of the solvent was kept constant throughout the extraction process.

#### 2.5.1. FRAP Assay

The ferric-reducing antioxidant power (FRAP) assay was performed according to the method described by Brito et al. [39]. To prepare the FRAP working solution, we combined acetate buffer (300 mmol L^−1^, pH 3.6), TPTZ (10 mmol L^−1^) dissolved in HCl (40 mmol L^−1^), and ferric chloride (20 mmol L^−1^) in a 10:1:1 (*v*/*v*/*v*) ratio. To create a calibration curve and determine the regression equation, we prepared Trolox solutions at a concentration of 5 mM with the following dilutions: 1.250, 0.900, 0.625, 0.313, 0.156, 0.078, and 0.039 mmol (y = 1.8529x ± 0.1436; R^2^ = 0.998).

For the assay, 20 μL of the sample, standard, or distilled water (as a blank) was added to each well of a 96-well microplate, followed by 180 μL of the prepared FRAP solution. The mixtures were agitated and incubated at 37 °C in the dark for 30 min. After incubation, the absorbance was measured at 593 nm using a microplate reader.

#### 2.5.2. DPPH Assay

The DPPH assay conducted in this study was based on the method outlined by Brito et al. [39]. To create the DPPH radical solution, a 35 mg DPPH standard solution in 10 mL of ethanol was prepared. Subsequently, the DPPH stock solution was made by diluting the standard solution in ethanol (70:30, *v*/*v*) to achieve an absorbance of 1.000 ± 0.04 at 520 nm.

To generate a calibration curve and regression equation, Trolox solutions (5 mM) were prepared at concentrations of 1.250, 0.900, 0.625, 0.313, 0.156, 0.078, and 0.039 mmol (y = 65.5596x ± 1.6577; R^2^ = 0.993). For the DPPH assay, 190 μL of the DPPH solution and 10 μL of the Trolox standard, sample, or distilled water (blank) were added to each microplate well. The mixture was incubated in the dark at room temperature for 30 min, and the absorbance was then measured at 520 nm. The percentage inhibition of the DPPH free radical can be calculated using the following formula:

% inhibition = 100 × (Abs_520_ blank − Abs_520_ sample)/Abs_520_ blank

The results were presented in μmol of Trolox per gram (μmol Trolox g^−1^).

#### 2.5.3. ABTS•+ Assay

The free radical scavenging activity of the ABTS radical was assessed using the method described by Brito et al. [39]. First, 88 μL of potassium persulfate (140 mmol L^−1^) was mixed with 5 mL of an ABTS (7 mmol L^−1^) solution. The mixture was subsequently stored in a covered bottle in the dark at room temperature for 16 h to generate the ABTS•+ radical cation. The absorbance of the ABTS solution was then adjusted to 0.70 ± 0.05 at 734 nm using a spectrophotometer.

Trolox solutions (1 mM) at concentrations of 0.140, 0.098, 0.069, 0.048, and 0.034 mmol were prepared to create a calibration curve and regression equation (y = 195.2165x ± 0.3092; R^2^ = 0.995). In the assay, 188 μL of the ABTS•+ solution and 12 μL of the sample or standard were added to each microplate well. A separate well containing 188 μL of ABTS•+ solution and 12 μL of distilled water served as the blank. The microplate was then incubated in the dark at room temperature for 2 h. After incubation, the absorbance was measured at 734 nm.

The results were reported as micromoles of Trolox equivalent per gram of sample (μmol Trolox g^−1^).

### 2.6. Determination of Tannin Content

The analyses performed for tannin content determination were the methylcellulose precipitable and condensed tannin assay. For all analyses, three replicates (*n* = 3) were performed in aliquots, and the absorbance measurements were taken with a UV-Vis spectrophotometer (Thermo Scientific^®^, model Evolution Pro, Vantaa, Finland).

#### 2.6.1. Methylcellulose Precipitable (MCP) Tannin Assay

To each sample, 1 g (± 0.04 g) was mixed with 10 mL of aqueous ethanol (50% *v*/*v*) and rotated for 60 min, followed by centrifugation at 4000 rpm for 5 min using a Sigma centrifuge (Steinheim, Germany). A 0.04% *w*/*v* methylcellulose solution was prepared according to the manufacturer’s instructions (Sigma-Aldrich, Castle Hill, NSW, Australia). Prior to analysis, the olive cake homogenate extracts were thawed at room temperature. The MCP (methylcellulose precipitable) tannin assay was carried out following the method described by Mercurio et al. [40] with slight modifications. For each sample, 1 mL of the supernatant was transferred into a 15 mL centrifuge tube, and 3 mL of methylcellulose solution or distilled water (as a control) was added. After a 3 min reaction, 2 mL of ammonium sulfate and 4 mL of distilled water were introduced. The mixture was vortexed and left to stand for 2 h before being centrifuged at 4000 rpm for 15 min using a Sigma centrifuge (Steinheim, Germany). Aqueous (−) epicatechin solutions at concentrations of 10, 25, 50, 75, 100, 150, 200, and 250 mg L^−1^ were used to create a calibration curve for tannin absorbances. Tannin values were reported in milligrams per liter (mg L^−1^) of epicatechin equivalents.

#### 2.6.2. Condensed Tannin Assay

The concentration of condensed tannins (proanthocyanidins) was measured using the vanillin reaction method described by Burns (1971) with minor modifications [41].

For extraction, 200 mg of the sample was weighed in triplicate (*n* = 3) and 10 mL of 1% HCl in methanol was added. The mixture was stirred for 1 h and then centrifuged at 4000 rpm for 20 min. Following centrifugation, 1 mL of the supernatant was transferred to a test tube and 2.5 mL of 1% vanillin solution in methanol and 2.5 mL of 8% HCl in methanol were added.

After 20 min, the absorbance was measured at 500 nm. The concentration of condensed tannins was calculated by constructing a calibration curve with catechin concentrations in methanol (0.50, 0.20, 0.15, 0.10, and 0.05 mg mL^−1^). The regression equation for the calibration curve was used, y = 0.3919x + 0.0125, R^2^ = 0.9905, where y represents absorbance and x is the catechin concentration. Results were expressed as milligrams of catechin equivalents per 100 g of sample (mg CAT/100 g).

### 2.7. Analysis of Individual Phenolic Compounds by High-Performance Liquid Chromatography (HPLC-PDA-MS)

The polyphenolic composition of the different OC samples was evaluated using HPLC-PDA-MS, following a previously described methodology [42]. Dehydrated OC samples (250 mg) were mixed with 1.5 mL of an ethanol/formic acid/water solution (50:2:48, *v*/*v*/*v*), vortexed, and sonicated in an ultrasonic bath for 60 min. The samples were then kept at 4 °C overnight and subjected to 60 min of sonication. Afterwards, the mixture was centrifuged at 10,000× *g* for 5 min to separate the extract from the solid residue. The supernatants were subsequently filtered through a 0.22 µm PVDF filter (Millex HV13, Millipore, Bedford, MA, USA).

Chromatographic separations were conducted using a Thermo Scientific VANQUISH C18 column (150 mm × 2.1 mm, 2.2 µm particle size; Thermo Fisher Scientific Inc., Vilnius, Lithuania). The phenolic profile was chromatographically resolved using deionized water/formic acid (99.9:0.1, *v*/*v*) (Solvent A) and acetonitrile/formic acid (99.9:0.1, *v*/*v*) (Solvent B) with the following gradient: (time, %B): (0, 10%), (20, 60%), (20.1, 10%), and (25, 10%). The flow rate was set to 0.3 mL/min with an injection volume of 7 µL. The HPLC system was equipped with a Vanquish–LTQ-XL–Thermo Scientific diode array and a mass detector in series (Thermo Scientific Dionex UltiMate 3000 Series, Germany). It included a quaternary SD, RS, BM, and BX pump, an autosampler, a degasser, and an electrochemical photodiode array detector, all controlled by Xcalibur software version 08.03 (Agilent Technologies, Waldbronn, Germany). Spectroscopic data from all peaks were collected within the range of 240–600 nm, and the spectral data were recorded at 280, 330, and 370 nm.

The mass detector was an ion trap mass spectrometer with an electrospray ionization (ESI) system controlled by LTQ Tune software version 4.1 (Agilent, Waldbronn, Germany). Nitrogen was used as the nebulizing gas at a pressure of 60 psi with a flow rate adjusted to 11 L/min. The heated capillary temperature and ionization voltage were maintained at 350 °C and 5 kV, respectively. Fragmentation experiments induced by collisions were carried out in the ion trap, utilizing helium as the collision gas, with voltage ramp cycles ranging from 0.3 to 2 V. The full scan mass covered a range from *m*/*z* 100 to *m*/*z* 2000, and mass spectrometry data were acquired in negative ionization mode. Total ion chromatograms were recorded as full-scan mass spectra (MS). The identification of individual phenolic compounds was performed by analyzing retention time (in minutes), parent ions, and fragmentation patterns, comparing them with authentic standards when available or utilizing descriptions from the literature.

## 3. Statistical Analysis

The regression equation (y = ax ± b), the coefficient of determination (R^2^), adjusted R^2^, and the correlation coefficient (r) were calculated for each method. The data were assessed for normality using the Shapiro–Wilk test and subjected to analysis of variance (ANOVA), followed by a multiple range test (Tukey’s test or *t*-test) with a significance level of *p* < 0.05, using JMP Statistics 17.1.0 software (JMP, Cary, NC, USA). Results were expressed as mean values ± standard deviation (*n* = 3).

To explore the relationships between phenolic composition, total and condensed tannins, and their antioxidant capacity, principal component analysis (PCA) was performed. PCA is a commonly used method for examining the strength of relationships between continuous variables, helping to identify the mathematical connections between response variables and determine the proportion of variation in one variable that can be predicted from another. The analysis and its statistical significance were conducted using JMP Statistics 17.1.0 software (JMP, Cary, NC, USA).

## 4. Results and Discussion

### 4.1. Phenolic Content, Tannin Content, and Antioxidant Capacity

As detailed in Table 1, EOC 2 exhibited the highest concentrations of total phenols (19.61 ± 5.61 mg GA/g dry weight (DW)), *ortho*-diphenols (21.82 ± 5.98 mg GA/g DW), and flavonoids (20.12 ± 5.54 mg CAT/g DW), showing significant differences (*p* < 0.05) compared to the other OCs. EOC 1 followed, with the second-highest levels of total phenols (12.31 ± 5.61 mg GA/g DW), while TPOC (partially pitted and dehydrated) averaged 7.20 mg CAT/g DW. COC 1 and COC 2 had the lowest flavonoid content at a mean of 4.14 mg CAT/g DW, with COC 2 significantly differing (*p* < 0.05) from COC 1, recording the lowest content among the cakes analyzed (5.08 ± 0.43 mg GA/g DW).

The total and condensed tannin contents of various olive cakes are also shown in the following table, highlighting significant variations. The highest total tannin contents were observed in the dehydrated and pitted TPOC (250.31 ± 0.20 and 240.89 ± 0.11, respectively), whereas COC 2 exhibited the lowest content (88.17 ± 0.05). COC 1, EOC 2, and EOC 1 were statistically similar to each other (*p* < 0.05), with the second-highest total tannin contents recorded (186.19 ± 0.13, 182.95 ± 0.25, and 155.06 ± 0.11, respectively).

In contrast, the condensed tannin content showed even greater variation across the different olive cakes. EOC 2 demonstrated the highest condensed tannin content (328.17 ± 0.06), while COC 2 displayed the lowest (20.56 ± 0.02). The dehydrated and ginned cakes were statistically similar to each other (117.80 ± 0.01 and 83.50 ± 0.07, respectively) and differed significantly from EOC 1 (48.91 ± 0.02). Additionally, EOC 1 (64.64 ± 0.05) exhibited statistically similar levels to both COC 1 and COC 2, which recorded the lowest values. These findings emphasize the importance of understanding the composition of different olive cake types to optimize their use as sources of bioactive compounds in various applications.

Valorizing OC, a by-product of olive oil production, can significantly reduce carbon footprints and increase the recovery of bioactive compounds with broad applications in industries such as food, pharmaceuticals, and energy. This approach is in line with sustainable development goals and provides both environmental and economic benefits [43,44].

The phenolic profile in olive cake can be influenced by several factors, including the origin of the olives, fruit ripeness, agronomic practices, soil and climate conditions during cultivation, the oil extraction process, and storage duration [45,46,47]. Researchers have explored various solvent extraction methods to optimize the recovery of phenolic compounds [48,49]. Traditional methods involve solvents like methanol, ethanol, and water in solid–liquid extraction processes to obtain phenolic-rich extracts [50].

Innovative extraction techniques such as microwave-assisted extraction (MAE), ultrasound-assisted extraction (UAE), electrotechnology, sub- and supercritical fluid extraction, and mechanical technologies have demonstrated effectiveness and environmental friendliness in extracting phenolic compounds [48,51]. These methods offer advantages in terms of efficiency, reduced extraction times, and minimized solvent use, making them attractive for sustainable extraction processes.

One study demonstrated that extraction with methanol at 70 °C for 12 h resulted in achieving the highest total phenolic content (4.37 mg/g) and antioxidant activity (73% inhibition) [52]. This method highlighted the effectiveness of prolonged methanol extraction at a moderate temperature in maximizing the extraction of phenolic compounds with potent antioxidant properties [53,54]. The findings from this study highlight significant variations in phenolic composition between different OC extracts, particularly between EOC 1 and EOC 2. EOC 2 exhibited a substantially higher phenolic compound content (19.61 ± 0.89 mg GAE/g) than EOC 1 (12.31 ± 0.52 mg GAE/g). This variation underscores the influence of extraction methods, olive varieties, and processing conditions on the quantification of phenolic compounds.

For instance, Martínez-Patiño et al. [55] reported a much higher total phenolic content (57.5 mg GAE/g) using ultrasound-assisted extraction with 43% ethanol. This disparity further emphasizes the impact of different extraction techniques, as varying solvent systems and extraction parameters can lead to significantly different results. In contrast, Goldsmith et al. [56] found a total phenolic content of 13.8 mg GAE/g in OC defatted with hexane, which aligns closely with our findings for EOC 1 but is notably lower than EOC 2. Defatting olive cake with hexane, a common practice to extract olive oil from pomace, can influence the remaining phenolic composition in the OC [50,57]. These observations underscore the importance of optimizing extraction methods tailored to specific goals, whether maximizing total phenolic content or targeting specific phenolic compounds with desirable bioactive properties. Understanding these factors is crucial for effectively valorizing olive cake as a rich source of bioactive compounds with potential applications in various industries.

The phenolic content, composition, and biological properties of olive oil (OC extracts) can vary due to several factors, including the olive variety, environmental conditions, geographic location, and extraction methods. As a result, directly comparing our findings with those in the existing literature can be challenging.

Tannins are distinguished by their ability to bind and precipitate proteins, with two main categories, namely hydrolysable tannins (like ellagitannins) and condensed tannins (proanthocyanidins). Widely distributed in foods such as grapes, nuts, and tea, tannins are renowned for their antioxidant, antimicrobial, and anti-inflammatory properties, contributing to various health benefits [58,59]. Tannins are among the most abundant phenolic compounds in the plant kingdom, following lignins [60]. These secondary metabolites play pivotal roles in plants, particularly defense mechanisms against stressors, highlighting their vital protective functions [61,62,63]. Tannins’ chemical diversity and functional versatility underscore their significance across different applications. Extracted from renewable sources, including olive by-products, tannins find applications in diverse fields, such as coagulants, adhesives, and colorants. The emphasis is on environmentally friendly extraction methods that replace synthetic compounds with natural alternatives, enhancing sustainability [61].

The analysis of total and condensed tannins in olive oil by-products presents challenges primarily because standardized quantitative methods are needed. Despite the availability of various protocols for quantification, such as colorimetric, chromatographic, and enzymatic techniques, these methods are only partially standardized across different matrices that require analysis [64,65]. The complexity and diversity of tannins necessitate reliable methods that yield comparable results across different tannins and account for chemical interactions between tannins/phenols and the reagents used [66].

Tannic acid is frequently used as a reference standard, but it differs biologically from flavonoid tannins prevalent in cereals and legumes. Its extraction is complicated by potential insolubility and polymerization with carbohydrates or proteins [67,68]. Therefore, ensuring an accurate and consistent quantification of tannins in olive by-products requires careful consideration of these methodological and chemical factors to obtain reliable analytical outcomes. Research into the chemical composition of OC, particularly its total and condensed tannin content, remains an active area of study. Previous investigations have primarily focused on evaluating the influence of tannins on nutrient digestibility. Yáñez-Ruiz and Molina-Alcaíde observed that incorporating a two-phase olive cake concentrate diet into sheep and goat diets increased the condensed tannin content in diets from 2.77 g/kg to 16.7 g/kg. They reported a condensed tannin content of 43.4 g/kg DM for the two-phase olive cake (TPOC) [69]. In our study using olive cake from a similar extraction system, we found a lower TPOC (pitted) value of 9.27 g/kg, while TPOC (dehydrated) showed a higher content of 33.53 g/kg. In another study by the same authors, they examined the chemical composition and nutrient availability of two-phase olive cake and olive leaves, reporting approximately 14 g/kg of condensed tannins for the pomace and 8.30 g/kg for the leaves [70]. These findings underscore the variability in condensed tannin levels influenced by factors such as olive pomace processing techniques and specific agricultural practices. This highlights the ongoing need for further research to establish comprehensive data on the chemical composition of olive pomace and explore its potential applications. Specific information on the chemical composition of various olive pomaces remains limited, although similarities may exist between those derived from two-phase and three-phase centrifugation extraction systems. Typically, pomace from the two-phase system is anticipated to be richer in polyphenols due to the inclusion of the aqueous extract that is separately obtained in the three-phase system. Consequently, higher tannin content is expected [70].

The variability in reported tannin content for similar OCs obtained from the same extraction technology can be attributed to differences in quantification methodologies and the influence of geographical, climatic, and sample storage conditions. These factors significantly impact results, often complicating direct comparisons with values found in the literature [71].

The phenomenon of higher condensed tannins (CTs) compared to total tannins (TTs) in the EOC 2 extract can be explained by the fact that the methods used to measure these compounds may target different aspects of tannin composition. Condensed tannins, which are oligomeric and polymeric phenolic compounds, may be more stable and less prone to degradation during the extraction and analysis processes compared to hydrolyzable tannins. This stability can lead to overestimating CT levels if the analytical methods do not fully account for the presence of hydrolyzable tannins. Also, the specific composition of the extract matrix could also influence the results, as other phenolic compounds may interfere with the measurements, affecting the total tannin quantification while allowing for a more accurate assessment of condensed tannin levels.

The antioxidant capacity of the six OC extracts was evaluated using three distinct methodologies. As summarized in Table 2, EOC 2 demonstrated significantly higher antioxidant capacity compared to the FRAP (129.98 ± 0.00 μmol Trolox/g), DPPH (78.00 ± 0.01 μmol Trolox/g), and ABTS (56.65 ± 0.00 μmol Trolox/g) assays. Notably, in the ABTS test, EOC 1 and COC 2 did not differ significantly from EOC 2 regarding antioxidant capacity. EOC 1 also exhibited strong results, ranking second in both FRAP (114.42 ± 0.01 μmol Trolox/g) and DPPH (56.14 ± 0.01 μmol Trolox/g) tests, as well as ABTS (56.65 ± 0.00 μmol Trolox/g) tests. In contrast, TPOC (pitted and dehydrated) showed statistically similar antioxidant capacities in the FRAP (48.39 and 45.45 ± 0.00 μmol Trolox/g, respectively) and DPPH (28.30 and 32.68 ± 0.00 μmol Trolox/g, respectively) assays. Both COC 1 and COC 2 displayed the lowest antioxidant capacities among the samples tested, scoring 32.61 ± 0.00 μmol Trolox/g in the FRAP test and 17.24 and 18.98 ± 0.00 μmol Trolox/g, respectively, in the DPPH test.

The reduction in antioxidant capacity followed a similar trend to total phenolic compounds. Phenolic compounds are recognized as the primary antioxidants in olive extracts. [72]. The overall antioxidant capacity of an extract reflects both “antiradical” and “antioxidant” activity, which do not always coincide. Antiradical activity measures the ability of compounds to react with free radicals (for example, in the ABTS and DPPH assays). In contrast, antioxidant activity represents the ability to inhibit oxidation processes (as in the FRAP assay) [73,74]. To better understand the overall antioxidant capacity of olives and olive oil, the methanolic extracts were evaluated using three different assays covering different antioxidant defense mechanisms, namely DPPH, ABTS radical scavenging activity, and FRAP. The influence of phenolic compounds on the antioxidant quality of fruit and olive oil is well established [72,75,76]. However, other compounds with antioxidant capacity are also present in these products and OC extracts and are positively correlated with their total polyphenol content [77,78].

The results indicate that the ABTS assay generally showed greater variability in the OC samples compared to the FRAP and DPPH assays. This variability can be attributed to factors such as the method’s sensitivity, the reactions’ kinetics and the complex nature of the phenolic compounds present in these samples [79]. Due to its characteristics, the ABTS assay interacts differently with various antioxidant compounds, especially with different phenolic compounds. For example, certain dihydrochalcones and flavanones, which do not react with the DPPH radical, can react with the ABTS radical, leading to notable differences in the antioxidant potentials measured. This lower sensitivity can lead to less precise measurements and greater variability in the results [79,80].

On the other hand, the FRAP assay, which evaluates the reducing power of antioxidants, tends to provide more consistent results in different samples, thanks to its direct and more straightforward reaction mechanism. Similarly, the DPPH assay, which determines radical scavenging activity, shows greater stability and less variability in its results. Traditional antioxidant standards such as Trolox and ascorbic acid exhibit stable and simple reaction kinetics with these assays, facilitating endpoint analysis and reducing variability [79].

Several authors have observed that extracts derived from OC have a significant antioxidant capacity, possibly related to the content of phenolic compounds [27,78,81,82]. For example, Ribeiro et al. [81] assessed the antioxidant capacity of OC extracts using three different methods (DPPH, ABTS, and FRAP), and their results indicated high antioxidant activity in OC methanolic extracts. Nunes et al. [82] also identified a relationship between antioxidant activity (measured by the DPPH, ABTS, and FRAP methods) and HT content in water-soluble extracts of OC by-products from various olive varieties from the northeastern (behind-the-hills) and southern (Alentejo) regions of Portugal.

### 4.2. Quantification and Identification of Individual Phenolic Compounds by High-Performance Liquid Chromatography (HPLC-PDA-MS)

The characterization of the individual phenolic compounds of the different olive cake extracts was determined using HPLC-PDA-MS methodology. Just as they are abundant in olive leaves, bioactive phenolic compounds can be recovered in olive by-products, including olive mill wastewater (OMWW) and in the pomace generated [4]. The most widely used analytical methods for identifying and quantifying bioactive compounds in olive oil by-products involve combining gas (GC) or liquid chromatography (LC) with mass spectrometry (MS). Mass spectrometry is the most effective and successful detection tool for providing detailed structural information. Without the need for derivatization, LC-MS, especially with electrospray ionization (ESI) or atmospheric pressure chemical ionization (APCI) sources, has become one of the most essential and versatile instrumental platforms for identifying bioactive phenolic compounds [83]. A summary of the bioactive compounds identified in the different olive cake extracts analyzed using MS techniques is reported in Table 3.

In our investigation, we were able to identify 22 compounds in total (in the six extracts analyzed) belonging to the classes of phenylethanoid glycosides (decaffeoyl verbascoside, β-hydroxy-isoverbascoside, verbascoside, and oxidized isoverbascoside), iridoids and iridoid glycosides (oleoside glucoside, oleoside 11-methl ester, oleoside diglucoside, caffeoyl-6′-secologanoside, loganic acid glucoside, and jaspolyoside derivatives), secoiridoids and derivatives (oleuropein glucoside isomers, oleuropein derivative 1, and oleuropein), flavones (luteolin-7-O-rutinoside, diosmin, apigenin-7-O-rutinoside, apigenin-7-O-glucoside, luteolin, and luteolin derivatives), phenolic compounds (hydroxytyrosol), phenolic acids (vanillic acid hexoside), and coumarins (aesculetin (dihydroxy coumarin isomers)). Of the main components of the phenolic fraction of the extracts analyzed, iridoids (27%), flavones (27%), phenylethanoid glycosides (18%), and secoiridoids (13%) were the most abundant, followed by phenolic compounds (5%), phenolic acids (5%), and finally coumarins (5%), which represent the smallest fraction of the compounds identified.

Cardoso et al. [84,85] analyzed methanolic extracts from olive pulp and OC, isolating 27 fractions that were subsequently examined using ESI-MS. Their analysis revealed several phenolic compounds common to both extracts, many of which are also typically found in olive leaves. These compounds include verbascoside, rutin, caffeoylquinic acid, luteolin-4-glucoside, 11-methyl-oleoside, hydroxytyrosol-1′-β-glucoside, luteolin-7-O-rutinoside, oleoside (along with its derivatives 6′-β-glycopyranosyl-oleoside and 6′-β-rhamnopyranosyl-oleoside), and 10-hydroxyoleuropein.

The main compounds identified include β-hydroxy-isoverbascoside, verbascoside, oleuropein glucoside isomers, oleuropein, luteolin, and vanillic acid hexoside, distributed among six extracts, which are EOC 1, EOC 2, TPOC (pitted), TPOC (dehydrated), COC 1, and COC 2. Cardinali et al. [86] successfully characterized and quantified verbascoside, isoverbascoside, and their derivatives using LC-DAD-MS/MS analysis combined with low-pressure gel filtration chromatography. Similarly, Obied et al. [87] validated this using RPLC-ESI-DAD-MS for analyzing olive mill waste (OMW), identifying 52 distinct compounds. These findings underscore the potential of olive oil industry by-products as a valuable reservoir of bioactive compounds such as oleacin, tyrosol, hydroxytyrosol, verbascoside, hydroxytyrosol glycoside, gallic acid, caffeic acid, luteolin, oleoside, oleuropein and its derivatives, rutin, and oleuroside [83].

Among the most common phenolic compounds in olives and their by-products, hydroxytyrosol was identified and quantified in only two of the six extracts analyzed, namely EOC 1 (4.72 mg HT/100 g) and EOC 2 (22.41 mg HT/100 g). Gómez-Cruz et al. [14], when analyzing the aqueous extract of EOC, obtained 6.3 HT/g EOC under optimized conditions. Hydroxytyrosol and tyrosol are compounds known for their high oxidative stability and potent antioxidant, anti-inflammatory, and antimicrobial activities. They are widely used as therapeutic agents, food supplements, and natural ingredients in the human and animal food industry [88,89,90]. According to Cardoso et al. [85], these compounds remain in the pomace even after oil extraction. However, the amount can vary considerably depending on the variety of olive trees, growing conditions, and oil extraction method [91]. For instance, Pérez-Serradilla et al. [91] identified 0.89 mg/g of HT in olive pomace using microwave-assisted extraction with methanol and hexane. Similarly, Habibi et al. [92] observed a maximum concentration of 1.57 mg/g, employing a comparable method combined with dispersive liquid–liquid microextraction. Conversely, Xie et al. [89] reported a much higher concentration of 49 mg/g of HT in olive pomace after ethanol extraction.

Castro et al. [68] comprehensively analyzed phenolic compounds in OC using a 50:50 (*v*/*v*) superheated water/ethanol mixture at 160 °C for extraction. Identification and validation of the compounds were achieved through accurate m/z ratios and isotopic patterns of precursor and product ions. Similarly, another study detected minor secoiridoids and phenolic compounds in *alperujo* extracts processed with polyamide and XAD resin through ESI-FTMS. The concentrated and purified extracts yielded three significant fractions enriched with minor compounds, including oleuropein derivatives such as oleoside methyl ester, dihydro-oleuropein, neo-nüzhenide, and oleuropein diglycoside, which were identified for the first time.

In contrast, EOC 2 only presented seven compounds. Although both OCs come from the same olive oil extraction system, they were collected in different years and at other mills. Most phenolic compounds found in olive oil by-products are susceptible to environmental conditions such as temperature, pH, and light, which could account for the observed variations. Recently, it has been demonstrated that the secoiridoids present in olive oil are highly sensitive to various factors, including the cultivar and geographical origin of the olive [93], the stage of ripeness during harvest, agronomic practices, and processing techniques [83,94]. Furthermore, storage conditions can significantly influence the preservation of these compounds [88]. These same factors are expected to impact the distribution and integrity of the phenolic compounds found in olive by-products.

Among these factors, it poses a significant challenge because the by-products of olive oil production are typically stored in tanks for extended periods, often spanning several months during the olive oil processing season [83]. Mattonai et al. [95] reported a substantial reduction in the phenolic content of olive mill wastewater following a one-year storage period.

When comparing the extracts of pitted olive cake (from two-phase centrifugal extraction) and dehydrated TPOC, there was a difference in the variety of compounds identified. The pitted TPOC had the lowest number of compounds identified, with only four compounds detected (19% of the total analyzed). On the other hand, the dehydrated TPOC had nine compounds identified (43% of the total analyzed). The difference can be explained by the specific distribution of bioactive compounds in the various parts of the olive plant [96].

**Figure 2:**
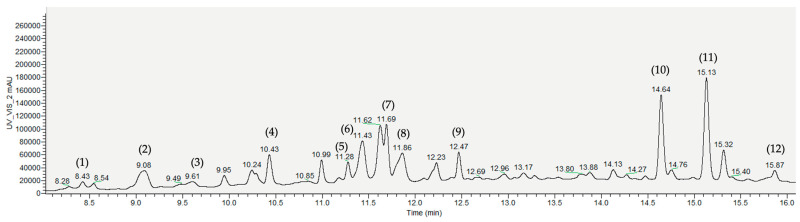
Representative HPLC-PDA-MS chromatograms (at 320 nm) of phenolic extracts obtained from crude olive cake (COC 2). Identified compounds: (1) β-hydroxy-isoverbascoside; (2) oleoside 11-methyl ester; (3) oleuropein glucoside isomers; (4) luteolin-7-O-rutinoside; (5) diosmin; (6) apigenin-7-O-rutinoside; (7) apigenin-7-O-glucoside; (8) caffeoyl-6′-secologanoside (cafselogoside); (9) oleuropein; (10) luteolin; (11) jaspolyoside derivative; (12) luteolin derivative.

The COC extracts also showed the most incredible diversity of phenolic compounds, specifically the COC 2 extract (Figure 2; 12 compounds identified). In both samples, flavones were the most predominant compounds.

The discrepancy in the phenolic profile of the different samples analyzed underscores the specific distribution of bioactive compounds across other parts of the olive plant. Compounds like salidroside, nuezhenide oleoside, and nuezhenide are predominantly found in the olive seed, while verbascoside is prevalent in both the pulp and seed. Other compounds such as tyrosol, hydroxytyrosol, oleuropein, and 3,4 DHFEA-EDA are distributed across various plant parts, including the pulp, leaves, and stones [96]. These findings highlight the importance of understanding the localization of bioactive compounds within the olive plant for optimizing extraction processes and maximizing the potential utilization of olive by-products in various industries.

### 4.3. Principal Component Analysis (PCA)

**Figure 3:**
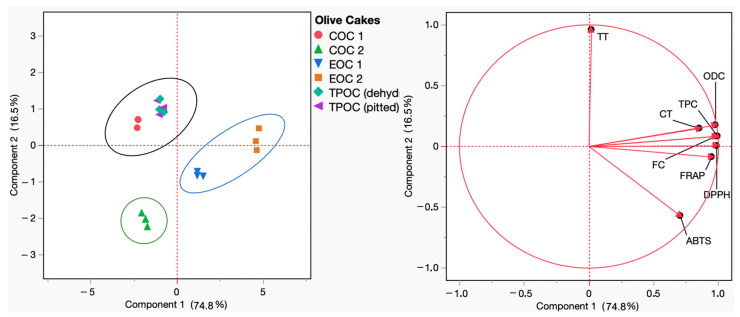
Principal component analysis (PCA) scores and loading plots of total phenol content (TPC), *ortho*-diphenol content (ODC), flavonoid content (FC), total tannins (TT), condensed tannins (CT), and the antioxidant capacity (FRAP, DPPH, and ABTS) of COC 1 (red), COC 2 (green), EOC 1 (blue), EOC 2 (orange), TPOC (dehydrated) (seafoam green), and TPOC (pitted) (purple). EOC: exhausted olive cake; TPOC: two-phase olive cake; COC: crude olive cake.

Principal component analysis (PCA) constitutes an essential mathematical approach for visualization and dimensionality reduction that transforms a high-dimensional data pool into an alternative of a lower dimension. Nonetheless, interestingly, this conversion preserves as much information as possible, resulting in new pools of data that allow for an understanding of the relationship between the (poly)phenolic content and the radical scavenging and reducing capacities of a given extract (e.g., the (poly)phenolic extracts of olive cake). According to the PCA results in Figure 3, the PC1 and PC2 components accounted for 74.8% and 16.5% of the loading score, respectively. Within the upper and lower right quadrants, a distinct grouping emerges, comprising the samples EOC1 and EOC2. This distribution agrees with the high values recorded for almost all parameters monitored (total phenolics, *ortho*-diphenols, flavonoids, DPPH• and ABTS•+ scavenging capacity, and FRAP) concerning olive cake samples. In contrast, COC 2 exhibited the opposite distribution, being located in the lower left quadrant concerning the lower values recorded for the parameters monitored. Concerning the remaining samples, COC 1, TPOC (dehydrated), and TPOC (pitted) are located in the higher left quadrant, related to the higher total tannins quantified in this sample.

## 5. Conclusions

Our comprehensive study delved into the antioxidant properties and polyphenolic profiles of OC extracts derived from different olive oil extraction systems, highlighting their potential as rich sources of bioactive compounds. We found that exhausted olive cake samples (EOC 1 and EOC 2) exhibited notably higher concentrations of total phenols, *ortho*-diphenols, and flavonoids, which corresponded to enhanced antioxidant capacities as assessed by FRAP, DPPH, and ABTS assays. The application of liquid chromatography was pivotal in identifying a diverse range of phenolic compounds, with crude OC extracts (COC 1 and COC 2) revealing the highest diversity in phenolic composition. Among the compounds identified, the most abundant belonged to the class of iridoids and secoiridoids, with the jaspolyoside derivative showing the highest concentration, specifically in sample COC 2 (329.02 mg/100 g).

Our research aimed to uncover the bioactive potential of olive cake and its implications for various industries, including cosmetics, food, and nutraceuticals. These findings underscore OC as a promising source of antioxidants, offering opportunities to develop natural products. Further scientific validation is essential to understand their safety profile and nutritional benefits, particularly in developing functional foods.

## Figures and Tables

**Table 1:** Total phenolic, *ortho*-diphenol, flavonoid, total tannin, and condensed tannin content of different olive cake extracts.

Olive Cakes	TPC	ODC	FC	TT	CT
	(mg GA/g DW)	(mg GA/g DW)	(mg CAT/g DW)	(mg CAT/100 g)	(mg EPI/100 g)
EOC 1	12.31 ± 0.52 ^b^	13.75 ± 0.09 ^b^	10.24 ± 0.77 ^b^	155.06 ± 0.11 ^b^	64.64 ± 0.05 ^cd^
EOC 2	19.61 ± 0.89 ^a^	21.82 ± 0.35 ^a^	20.12 ± 0.31 ^a^	182.95 ± 0.25 ^b^	328.17 ± 0.06 ^a^
TPOC (pitted)	9.32 ± 0.51 ^c^	9.32 ± 0.60 ^c^	5.00 ± 0.39 ^c^	240.89 ± 0.11 ^a^	117.80 ± 0.01 ^b^
TPOC (dehydrated)	9.72 ± 0.19 ^c^	9.72 ± 0.22 ^c^	5.62 ± 0.32 ^c^	250.31 ± 0.20 ^a^	83.50 ± 0.07 ^bc^
COC 1	7.91 ± 0.39 ^d^	7.91 ± 0.61 ^d^	5.16 ± 0.13 ^c^	186.19 ± 0.13 ^b^	48.91 ± 0.02 ^c^
COC 2	5.08 ± 0.23 ^d^	5.08 ± 0.43 ^e^	2.76 ± 0.19 ^d^	88.17 ± 0.05 ^c^	20.56 ± 0.02 ^d^

EOC: exhausted olive cake; TPOC: two-phase olive cake; COC: crude olive cake; CAT: catechin; EPI: epicatechin; DW: dry weight; FC: flavonoid content; GA: gallic acid; ODC: *ortho*-diphenol content; TPC: total phenol content; TT: total tannins; CT: condensed tannins (*n* = 3 per olive cake). Data are presented as mean ± SD. Different letters in the same column correspond to significant differences between species (*p* < 0.05) using an ANOVA followed by a post hoc Tukey’s test.

**Table 2:** Antioxidant capacity of different olive cake extracts using FRAP, DPPH, and ABTS methods.

Olive Cakes	FRAP	DPPH	ABTS
	(μmol Trolox/g)	(μmol Trolox/g)	(μmol Trolox/g)
EOC 1	114.42 ± 3.35 ^b^	56.14 ± 4.76 ^b^	53.00 ± 1.20 ^a^
EOC 2	129.98 ± 4.36 ^a^	78.00 ± 4.52 ^a^	56.65 ± 0.16 ^a^
TPOC (pitted)	48.39 ± 2.98 ^c^	28.30 ± 1.00 ^c^	42.78 ± 0.98 ^b^
TPOC (deydrated)	45.45 ± 1.89 ^c^	32.68 ± 2.08 ^c^	44.47 ± 2.47 ^b^
COC 1	32.61 ± 0.10 ^d^	17.24 ± 1.34 ^d^	33.43 ± 1.05 ^c^
COC 2	32.61 ± 0.11 ^d^	18.98 ± 0.78 ^d^	52.55 ± 4.18 ^a^

EOC: exhausted olive cake; TPOC: two-phase olive cake; COC: crude olive cake; ABTS: scavenging capacity of the ABTS radical; DPPH: scavenging capacity of the DPPH radical; FRAP: ferric-reducing antioxidant power (*n* = 3 per olive cake). Data are presented as mean ± SD. Different letters in the same column correspond to significant differences between species (*p* < 0.05) using an ANOVA followed by a post hoc Tukey’s test.

**Table 3:** Quantification of phenolic compounds present in olive cake extracts by HPLC-PDA-MS.

Compound Id	RT	λ (UV)	*m/z*	Fragments	EOC	EOC	TPOC	TPOC	COC 1	COC 2
	(min)	(nm)	[M–H]^−^		1	2	(Pitted)	(Dehydrated)		
**Phenylethanoid Glycosides**										
**Decaffeoyl verbascoside**	3.60	280	461	375,123	×	N.D.	N.D.	N.D.	N.D.	N.D.
**β-hydroxy-isoverbascoside**	8.43	320	639	609,150	×	×	N.D.	×	N.D.	×
**Verbascoside**	10.46	320	623	477,153	×	×	×	N.D.	×	×
**Oxidized isoverbascoside**	11.08	320	621	609	N.D.	N.D.	×	×	N.D.	N.D.
**Iridoids and Iridoid Glycosides**										
**Oleoside glucoside** ^B^	5.98	280	551	389,191	63.97 ± 0.00 ^a^	N.D.	N.D.	N.D.	N.D.	N.D.
**Oleoside 11-methyl ester** ^B^	9.07	320	151			N.D.	N.D.	N.D.	N.D.	45.49 ± 1.03 ^a^
**Oleoside diglucoside** ^B^	9.75	320	713	477	60.45 ± 0.00 ^a^	N.D.	N.D.	N.D.	N.D.	N.D.
**Caffeoyl-6′-secologanoside** (cafselogoside) ^B^	11.84	320	553	431,285	N.D.	N.D.	N.D.	N.D.	14.73 ± 0.42 ^a^	N.D.
**Loganic acid glucoside** ^B^	12.97	320	537		N.D.	N.D.	N.D.	N.D.	L.I.	N.D.
**Jaspolyoside derivative** ^B^	15.16	320	909		N.D.	N.D.	N.D.	203.12 ± 3.91 ^b^	N.D.	329.02 ± 2.86 ^a^
**Total**								203.12 ± 3.91	14.73 ± 0.42	374.51 ± 1.95
**Secoiridoids and Derivatives**										
**Oleuropein glucoside isomers** ^B^	10.58	320	623	477,465	11.70 ± 0.00 ^d^	35.69 ± 0.30 ^b^	N.D.	39.74 ± 0.02 ^a^	6.42 ± 0.01 ^e^	26.54 ± 0.03 ^c^
**Oleuropein derivative 1** ^B^	10.95	320	543		60.91 ± 0.00 ^a^	N.D.	N.D.	N.D.	N.D.	N.D.
**Oleuropein** ^A^	12.5	320	539		N.D.	36.26 ± 1.80 ^c^	N.D.	193.54 ± 0.02 ^a^	L.I.	81.56 ± 2.74 ^b^
**Total**					72.61 ± 0.00	71.95 ± 1.05		233.28 ± 0.02	6.42 ± 0.01	108.1 ± 1.39
**Flavones**										
**Luteolin-7-O-rutinoside** ^C^	10.44	320	593	193	N.D.	N.D.	N.D.	3.73 ± 0.03 ^a^	N.D.	1.29 ± 0.04 ^b^
**Diosmin** ^C^	11.32	320	607	509	N.D.	N.D.	N.D.	N.D.	N.D.	0.65 ± 0.00 ^a^
**Apigenin-7-O-rutinoside** ^C^	11.42	320	577	549	N.D.	N.D.	N.D.	2.57 ± 0.01 ^a^	0.70 ± 0.01 ^c^	1.66 ± 0.04 ^b^
**Apigenin-7-O-glucoside** ^C^	11:69	320	431		N.D.	N.D.	N.D.	N.D.	N.D.	3.62 ± 0.26 ^a^
**Luteolin** ^C^	14.70	320	285		2.45 ± 0.03 ^e^	4.09 ± 0.02 ^c^	6.78 ± 0.01 ^b^	14.42 ± 0.00 ^a^	1.91 ± 0.01 ^f^	4.07 ± 0.10 ^d^
**Luteolin derivative** ^C^	15.86	320	615	329.201	N.D.	N.D.	N.D.	N.D.	N.D.	0.19 ± 0.02 ^a^
**Total**					2.45 ± 0.03	4.09 ± 0.02	6.78 ± 0.01	20.71 ± 0.01	2.61 ± 0.01	11.48 ± 0.08
**Phenolic Compounds**										
**Hydroxytyrosol** ^A^	10.15	320	153		4.72 ± 0.00 ^b^	22.41 ± 0.00 ^a^	N.D.	N.D.	N.D.	N.D.
**Total**					4.72 ± 0.00	22.41 ± 0.00				
**Phenolic Acids**										
**Vanillic acid hexoside** ^A^	15.88	320	329	201	0.40 ± 0.00 ^d^	1.14 ± 0.00 ^c^	2.72 ± 0.00 ^a^	0.23 ± 0.00 ^e^	1.71 ± 0.00 ^b^	N.D.
**Total**					0.40 ± 0.00	1.14 ± 0.00	2.72 ± 0.00	0.23 ± 0.00	1.71 ± 0.00	N.D.
**Coumarins**										
**Aesculetin (dihydroxhycoumarin isomers)**	11.30	320	241	177	N.D.	N.D.	N.D.	N.D.	×	N.D.

RT: retention time; ×: identified compound; N.D.: not detected; LI: low intensity; EOC: exhausted olive cake; TPOC: two-phase olive cake; COC: crude olive cake. Content is expressed as mg of phenol/100 g of lyophilized extract. Values are mean ± standard deviation from duplicate analyses. Different letters in the same column correspond to significant differences between species (*p* < 0.05) using an ANOVA followed by a post hoc Tukey’s test. ^A^ Compounds were identified and quantified with commercial standards; ^B^ compounds were quantified with a calibration of oleuropein; ^C^ compounds were quantified with a calibration of apigenin-7-glucoside.

## Data Availability

Data are contained within the article.
